# The *T*-Odd R and D Correlations in Beta Decay

**DOI:** 10.6028/jres.110.070

**Published:** 2005-08-01

**Authors:** Peter Herczeg

**Affiliations:** Los Alamos National Laboratory, Theoretical Division, Los Alamos, NM 87545, USA

**Keywords:** beta decay, CP-violation, physics beyond the Standard Model, time-reversal violation

## Abstract

We review and discuss the time-reversal-odd *R* and *D* correlations in neutron and nuclear beta decay.

## 1. Introduction

CP-violation (CPV)^1^ has been seen in the mixing of the neutral kaons, and recently also in the *K*° → 2π amplitudes [2] and in the decays of the neutral *B*-mesons [3]. At present there is no unambiguous direct evidence for time-reversal (T) violation.^2^ We know however that T-invariance is violated, since the parameter *ε* in *K*_L_ → 2π decays is dominated by a CPT-invariant interaction.^2^ In the models which we shall consider in the following all the interactions are CPT invariant, and we shall use therefore the terms “T-violation” and “CP-violation” interchangeably.

To date there is no firm evidence against the possibility that the observed CPV effects are due to the Kobayashi-Maskawa phase *δ_KM_* in the Standard Model (SM).^3^,^4^ A major question in the field of CPV is whether there are sources of CPV other than *δ_KM_*, independently of their relevance or lack of it for the observed CPV. New sources of CPV are present in many extensions of the SM. It is relevant to mention in this connection that *δ_KM_* is not sufficient to generate the baryon asymmetry of the universe.^5^ The most suitable observables to probe the existence of new CPV interactions are those for which the contribution from *δ_KM_* is small. Examples of observables of this kind are the electric dipole moments of the neutron and atoms, and T-odd correlations in leptonic and semileptonic decays.

In this talk we shall review and discuss the status of T-odd correlations in beta decay. In the next section we review the expressions for the coefficients of *D* and *R* correlations for a general $d\to ue-{\bar{\nu }}_{e}$ interaction. In Section 3 we summarize the limits on the CPV beta decay coupling constants implied by beta decay experiments. In Section 4 we consider *D* and *R* in extensions of the SM. Section 5 contains a summary of our conclusions.

## 2. General Considerations

Time-reversal (T) violating components in the $d\to ue-{\bar{\nu }}_{e}$ interaction manifest themselves in beta decay through contributions to T-odd correlations in the decay probability [9]. Sensitive experimental information is available on the coefficients *D* and *R* of the correlations 〈***J***〉 **· *p***_e_ × ***p****_ν_* / *J E*_e_ *E_ν_* and ***σ* ·** 〈***J***〉 × ***p***_e_ / *J E*_e_ (***σ*** ≡ electron spin, ***J*** ≡ nuclear spin, ***p***_e_ ≡ electron momentum, ***p****_ν_* ≡ neutrino momentum, *E*_e_ ≡ electron energy, *E_ν_* ≡ neutrino energy), respectively. The T-odd correlations are present even in the absence of T-violation, induced by final state interactions. The latter are dominated by contributions from the electromagnetic interaction. We shall write *D* and *R* as *D* = *D_t_* + *D_f_* and *R* = *R_t_* + *R_f_*, where *D_t_*, *R_t_* represent the T-violating contribution, and *D_f_*, *R_f_* are the T-invariant contributions due to the final state interactions.

In the SM the $d\to ue-{\bar{\nu }}_{e}$ transition arises from *W*-exchange, and has a V-A form:^6^

$$H=\left(G_{F}V_{ud}/\sqrt{2}\right)\bar{e}\gamma_{\lambda }\left(1-\gamma_{5}\right)\nu_{e}^{\left(L\right)}\bar{u}\gamma^{\lambda }\left(1-\gamma_{5}\right)d+H.c.,$$ (1)

where $G_{F}/\sqrt{2}=g^{2}/8M_{W}^{2}$, and *V_ud_* is the *ud*-element of the Kobayashi-Maskawa matrix. The neutrino state $\nu_{e}^{\left(L\right)}$ accompanies the left-handed electron in a doublet of *SU*(2)*_L_*. It is a linear combination of the left-handed components of the mass eigenstates:

$$\nu_{e}^{\left(L\right)}=\sum_{i}U_{ei}^{\left(L\right)}\nu_{iL},$$ (2)

where *ν_i_*_L_ = ½(1 − *γ*_5_)*ν_i_*.

The interaction (1) is CP- (and T-) invariant. In the quark and gluon sector of the SM there are two sources of CP-violation: the Kobayashi-Maskawa phase *δ_KM_* in the quark mixing matrix, and the *θ* -term in the QCD Lagrangian. *D_t_* and *R_t_* from these sources are extremely small, of the order of 10^−12^*a* [11], where *a* is defined in Eq. (10) below. The reason is that *δ_KM_* contributes only in second order in the weak interaction, and the *θ*-term is constrained by the stringent bound |*θ* | ≲ 4 × 10^−10^ from the experimental limit on the electric dipole moment of the neutron. In the SM with massive neutrinos CP-violation can be present also in the mixing of leptons. The effect of this in beta decay would not show up in first order in the weak interaction either. Thus *D_t_* and *R_t_* probe sources of CP-violation beyond those present in the SM.

To first order in new $d\to ue-{\bar{\nu }}_{e}$ interactions *D_t_* and *R_t_* arise from interference between the SM amplitude and the amplitude from the new interactions. We shall neglect in *D_t_* and *R_t_* terms proportional to neutrino masses. All the remaining terms must come from interactions involving left-handed neutrinos. The most general $d\to ue-{\bar{\nu }}_{e}^{\left(L\right)}$ interaction involving the neutrino state (2)^7^ can be written as 8

$$H_{\beta }^{\left(L\right)}=H_{V,A}^{\left(L\right)}+H_{S}^{\left(L\right)}+H_{P}^{\left(L\right)}+H_{T}^{\left(L\right)},$$ (3)

where

$$\begin{array}{l} H_{V,A}^{\left(L\right)}=\bar{e}\gamma^{\lambda }(1-\gamma_{5})\nu_{e}^{\left(L\right)}\\ \;\left[a_{LL}\bar{u}\gamma_{\lambda }(1-\gamma_{5})d+a_{LR}\bar{u}\gamma_{\lambda }(1+\gamma_{5})d\right]+H.c., \end{array}$$ (4)

$$H_{S}^{\left(L\right)}=a_{LS}\bar{e}(1-\gamma_{5})\nu_{e}^{\left(L\right)}\bar{u}d+H.c.,$$ (5)

$$H_{P}^{\left(L\right)}=a_{LP}\bar{e}(1-\gamma_{5})\nu_{e}^{\left(L\right)}\bar{u}\gamma_{5}d+H.c.,$$ (6)

$$H_{T}^{\left(L\right)}=a_{LT}\bar{e}\sigma_{\lambda \mu }\frac{1}{\sqrt{2}}(1-\gamma_{5})\nu_{e}^{\left(L\right)}\bar{u}\frac{1}{\sqrt{2}}\sigma^{\lambda \mu }d+H.c.,$$ (7)

The fields *e*, *u*, and *d* in Eqs. (4) – (7) are the mass eigenstates. The coupling constants are in general complex, in which case the Hamiltonians violate T-invariance. The constant *a_LL_* in Eq. (4) contains the SM contribution, and can therefore be written as, *a_LL_* = (*a_LL_*)*_SM_* + *a′_LL_*, where ${\left(a_{LL}\right)}_{SM}=g^{2}V_{ud}/8M_{W}^{2}$ and *a′_LL_* represents contributions from new interactions.

The contribution of the Hamiltonian, Eq. (3) to *D_t_* and *R_t_* in allowed beta decays is given by [9]

$$D_{t}≃aIm{\bar{a}}_{LR},$$ (8)

$$R_{t}≃\frac{a\mp b}{2g_{A}}g_{T}Im{\bar{a}}_{LT}-\frac{a}{2g_{V}}g_{S}Im{\bar{a}}_{LS},$$ (9)

where the upper (lower) sign in the first term in Eq. (9) is for decays with e^–^(e^+^) in the final state. In Eqs. (8) and (9) *ā_ik_* = *a_ik_/a_LL_* (*ik* = *LR*, *LT*, *LS*); *a* and *b* are constants containing the Fermi and Gamow-Teller matrix elements *M_F_* and *M_GT_*:

$$a=\frac{4\delta_{J\prime J}M_{F}M_{GT}{\left[J/(J+1)\right]}^{1/2}g_{V}g_{A}}{g_{V}^{2}{\left|M_{F}\right|}^{2}+g_{A}^{2}{\left|M_{GT}\right|}^{2}},$$ (10)

$$b=\frac{4\lambda_{J\prime J}{\left|M_{GT}\right|}^{2}g_{A}^{2}}{g_{V}^{2}{\left|M_{F}\right|}^{2}+g_{A}^{2}{\left|M_{GT}\right|}^{2}}.$$ (11)

In Eq. (11) *λ_J′J_* is an angular momentum factor, defined in Ref. [9]. The quantities *g_k_* ≡ *g_k_*(0) (*k* = *V*, *A*, *S*, *T*) are defined by

$$\langle p\left|\bar{u}\gamma_{\lambda }d\right|n\rangle =g_{V}\;\left(q^{2}\right){\bar{u}}_{p}\gamma_{\lambda }u_{n},$$ (12)

$$\langle p\left|\bar{u}\gamma_{\lambda }\gamma_{5}d\right|n\rangle =g_{A}\;\left(q^{2}\right){\bar{u}}_{p}\gamma_{\lambda }\gamma_{5}u_{n},$$ (13)

$$\langle p\left|\bar{u}d\right|n\rangle =g_{S}\;\left(q^{2}\right){\bar{u}}_{p}u_{n},$$ (14)

$$\langle p\left|\bar{u}\sigma_{\lambda \mu }d\right|n\rangle =g_{T}\;\left(q^{2}\right){\bar{u}}_{p}\sigma_{\lambda \mu }u_{n}.$$ (15)

CVC predicts *g_V_* = 1, and (neglecting the effects of the possible new interactions) the experimental value of *g_A_/g_V_* is *g_A_/g_V_* = 1.2670 ± 0.0030 [13]. The constants *g_S_* and *g_T_* were calculated in Ref. [14] in connection with a study of neutral current interactions of a general Lorentz structure. Employing a quark model with spherically symmetric wave functions, *g_S_* and *g_T_* are given by *g_S_* = − ^1^*/*_2_ + ^9^*/*_10_*g_A_* ≃ 0.6, *g_T_* = ^5^*/*_3_ (^1^*/*_2_ + ^3^*/*_10_*g_A_*) ≃ 1.46. The uncertainty in these predictions has been estimated to be about 30 % to 60% [14]. Including an uncertainty of this size, one has

$$0.25≲g_{S}≲1,$$ (16)

$$0.6≲g_{T}≲2.3.$$ (17)

For neutron decay *M_F_* = 1, $M_{GT}=\sqrt{3}$, implying *a* ≃ 0.87, *b* ≃ 2.2, so that

$$(D_{t})≃0.87Im{\bar{a}}_{LR},$$ (18)

$$(R_{t})≃-0.53g_{T}Im{\bar{a}}_{LT}-0.44g_{S}\;Im{\bar{a}}_{LS}.$$ (19)

## 3. Limits on the CP-Violating Coupling Constants From Beta Decay Experiments

The best current limits on $Im{\bar{a}}_{LR}$, and $Im{\bar{a}}_{LS}$, $Im{\bar{a}}_{LS}$ from beta decay experiments are

$$\left|Im{\bar{a}}_{LR}\right|<1.1\times 10^{-3}\;(90\%c.l.),$$ (20)

$$\left|g_{T}Im{\bar{a}}_{LT}\right|<8.6\times 10^{-3}\;(90\%\;c.l.),$$ (21)

$$\left|g_{S}Im{\bar{a}}_{LS}\right|≲0.1.$$ (22)

The limit, Eq. (20), follows from the result (*D*)_Ne_ = (0.1 ± 0.6) × 10^−3^ of a measurement of *D* in ^19^Ne decay [15]. For this decay *a* ≃ −1.03[15]. *D_f_* has been estimated to be *D*_f_ ≃ 2×10^−4^ *p*_e_(*p*_e_)_max_ [16]. Experiments to measure *D* in neutron decay are in progress at NIST by the emiT collaboration [17] and at the ILL by the Trine collaboration [18]. *D*_f_ is smaller in neutron decay than in ^19^Ne by an order of magnitude [16]. The initial run of the emiT experiment yielded (*D*)*_n_* = [0.6 ± 1.2(stat) ± 0.5(syst)] × 10^−3^ [19], implying | $Im{\bar{a}}_{LR}$| < 3.1 × 10^−3^ (90 % c.l.). The Trine experiment obtained (*D*)*_n_* = [−2.8 ± 6.4(stat) ± 3.0(syst)] × 10^−4^ [20], yielding | $Im{\bar{a}}_{LR}$| < 1.7 × 10^−3^ (90 % c.l.). Improved measurements of (*D*)*_n_* by the emiT and Trine collaborations are under way [17], [18].

The limit in Eq. (21) on the tensor interaction comes from the result (*R*_Li_)_expt_ = (1.6 ± 2.2) × 10^−3^ [21] of a measurement of *R* in ^8^Li → ^8^Be + e^–^ + *ν*_e_ decay. For this case one has *a* ≃ 0, and *b* = 4 / 3, so that *R_t_* ≃ −0.53 *g_T_ Imā_LT_*. Subtracting from (*R*_Li_)_expt_ the final state interaction contribution, which for this case is *R*_f_ ≃ 7 × 10^−4^ [21], yields *R_t_* = (0.9 ± 2.2) × 10^−3^ [21].

Finally, the limit, Eq. (22), follows from a measurement of the *e* – *ν* correlation in ^32^Ar beta decay [22]. A limit, which is weaker than (22), is implied by a measurement of *R* in ^19^Ne decay [23]. An experiment to measure *R* in neutron decay to an accuracy of 5 × 10^−3^ is being developed at PSI [21]. In neutron decay *R*_f_ ≃ 10^−3^. As seen from Eq. (19), such a result, combined with the bound in Eq. (21) will set an upper bound of about 2 × 10^−2^ on |*g_S_Imā_LS_*|.

## 4. *D_t_* and *R_t_* in Extensions of the Standard Model

In this section we shall discuss briefly *D_t_* and *R_t_* in extensions of the SM. We shall restrict our attention only to models where the required interactions can arise at the tree level, since loop-induced interactions are expected to be weak.

### 4.1 *D_t_*

An *a_LR_*-type interaction can arise at the tree level in models containing a new charged gauge boson with right-handed couplings to the quarks (as in left-right symmetric models), in the SM model if it is extended to contain new heavy “exotic” quarks which have right-handed couplings to the *W* and which mix with the known quarks, and in models with leptoquarks.^9^ In all these cases the *a_LR_*-interaction can be represented for beta decay by contact nonderivative four-fermion interactions. Contact *a_LR_*-interactions can arise also in composite models, from the exchange of constituents.^10^

Since the *a_LR_*-interaction is not invariant under the standard electroweak gauge group, it must be proportional to an *SU*(2)*_L_* × *U*(1) breaking parameter. In left-right symmetric models this is the nondiagonal element of the *W_L_-W_R_* mixing matrix, and in exotic fermion models the light-heavy quark mixing angles. In leptoquark models the *a_LR_*-interaction arises from mixing of leptoquarks of different SM quantum numbers. In composite models an *a_LR_*-interaction must contain the factor *υ/Λ* relative to the *SU*(2)*_L_* × *U*(1) invariant interactions, where *υ* is the vacuum expectation value of the SM Higgs boson and *Λ* is the compositness scale.

In left-right and exotic fermion models an *a_LR_*-type $d\to ue-{\bar{\nu }}_{e}$ interaction is accompanied by a strangeness conserving quark-quark interaction of strength *a_LR_*, which has a *P,T*-violating component of the form [26], [12]

$$H_{P,T}=-(Ima_{LR}){\left\{\bar{u}\gamma_{\lambda }(1+\gamma_{5})d,d\gamma^{\lambda }(1-\gamma_{5})u\right\}}_{+}+H.c.$$ (23)

The interaction (23) contributes to the electric dipole moment (EDM) of the neutron and to the isovector *P,T*-violating π*NN* coupling constant ${\bar{g}}_{\pi NN}^{(1)\prime }$. The latter induces atomic EDMs through the Schiff moment. The coupling constant ${\bar{g}}_{\pi NN}^{(1)\prime }$, which is given by the *N* → *N*π matrix element of the Hamiltonian (23), can be written as

$${\bar{g}}_{\pi NN}^{(1)\prime }=G_{F}V_{ud}m_{\pi }^{2}k(Im{\bar{a}}_{LR}),$$ (24)

where the constant *k* is expected to be of the order of *m*_π_/(*m_u_* + *m_d_*) ≃ 10, in view of the left-right structure of the operator, Eq. (23). The EDMs set stringent limits on $Im{\bar{a}}_{LR}$. The best one is

$$\left|Im{\bar{a}}_{LR}\right|≲\frac{5\times 10^{-5}}{k},$$ (25)

implied by the experimental upper limit (|*d*(^199^Hg)| < 2.1 × 10^−28^ e cm (90 % c.l.) [27]) on the EDM of the mercury atom.^11^ An estimate of *k* [30] using factorization and QCD sum rules yielded *k* ≃ 10, implying

$$\left|Im{\bar{a}}_{LR}\right|≲5\times 10^{-6}.$$ (26)

The neutron EDM, estimated in Ref. [30], leads to the limit $\left|Im{\bar{a}}_{LR}\right|≲10^{-5}$, nearly the same as Eq. (26).

For *D_t_/a* from leptoquark exchange the constraints are weaker [31]. The *P,T*-violating strangeness conserving quark-quark interaction, which is generated at one-loop level from diagrams involving *W*-exchange and containing a leptoquark propagator in one of the vertices, is suppressed by $m_{u}^{2}$ or $m_{d}^{2}$. The electron EDM and the quark electric and chromoelectric dipole moments do not arise at the one loop level. Based on dimensional estimates of the dipole moments, the conclusion is that they allow *D_t_/a* to be as large as the present experimental limit on *D_t_/a*.

### 4.2 *R_t_*

Scalar $d\to ue-{\bar{\nu }}_{e}$ interactions can arise at the tree level from the exchange of Higgs bosons, spin-zero or spin-one leptoquarks, and in supersymmetric models with *R*-parity violation from the exchange of sleptons. Tensor type $d\to ue-{\bar{\nu }}_{e}$ interactions can arise from the exchange of spin-zero leptoquarks. Scalar and tensor $d\to ue-{\bar{\nu }}_{e}$ interactions can appear also in composite models, generated by the exchange of constituents.

Let us consider *R_t_* in the minimal supersymmetric standard model with *R*-parity violation [32].

In the minimal supersymmetric standard model (MSSM), unlike in the SM, the conservation of lepton number (*L*) and of baryon number (*B*) is not automatic: the superpotential can contain renormalizable and gauge invariant *L*- and *B*-violating terms. If both the *L*- and the *B*-violating terms are present, some of the products of the corresponding coupling constants would have to be extremely small to prevent too rapid proton decay. One way to deal with this problem is to demand invariance under *R*-parity [R = (−1)^3^*^B^*^+^*^L^*^+ 2^*^s^*, where *s* is the spin of the particle; thus *R* = +1 for particles of the SM, and *R* = −1 for their superpartners]. This would eliminate both the *B*- and the *L*-violating terms. Alternatively, one can demand invariance under “baryon parity” (under baryon parity the quark fields change sign, and the lepton and Higgs fields remain unchanged), which eliminates only the *B*-violating terms. The model we shall consider in the following is the *R*-parity violating minimal supersymmetric standard model (RMSSM), defined as the MSSM with the lepton-number violating terms WL included in the superpotential.^12^

The general form of W_Ł_ is given by

$$W_{Ł}=\frac{1}{2}\lambda_{ijk}{L^{\prime }}_{i}{L^{\prime }}_{j}E_{k}^{c^{\prime }}+{\lambda^{\prime }}_{ijk}{L^{\prime }}_{i}{Q^{\prime }}_{j}D_{k}^{c^{\prime }}+\mu_{i}{L^{\prime }}_{i}H_{u},$$ (27)

where *i, j, k* = 1, 2, 3 are family indices, and summations over *i, j, k* are implied. In Eq. (27), ${L^{\prime }}_{i}$, ${Q^{\prime }}_{i}$ are the *SU*(2)-doublet lepton and quark superfields, $E_{i}^{c^{\prime }}$, $U_{i}^{c^{\prime }}$, $D_{i}^{c^{\prime }}$ are the *SU*(2)-singlet charged lepton and up- and down-type quark superfields; *H_u_* is the Higgs superfield which generates the masses of the up-type quarks. The primes on the fields indicate that they are the weak eigenstates.

The presence of *R*-parity violating couplings has rich phenomenological implications. One of these is that they can contribute to SM processes through the exchange of single squarks or sleptons.

There are two classes of contributions to beta decay. One of them is governed by ${\left|{\lambda^{\prime }}_{11k}\right|}^{2}$ and mediated by the ${\tilde{d}}_{kR}(k=1,2,3)$ [36]. These $d\to ue-{\bar{\nu }}_{e}$ interactions have a *V* − *A* form [36], and therefore do not contribute to T-odd correlations. The other class, which involves both *λ_ijk_* and *λ^′^_ijk_*, has scalar and pseudoscalar components. There are two such contributions, given by

$$H_{\beta }^{\left(j\right)}=\frac{\lambda_{1j1}{\lambda^{\prime }}_{j11}^{*}\omega_{B}}{4m_{{\tilde{e}}_{j}L}^{2}}\bar{e}(1-\gamma_{5})\nu_{1}\bar{u}(1+\gamma_{5})d+H.c.\;(j=2,3).$$ (28)

In the Hamiltonian, Eq. (28), the fields are the mass eigenstates; in the sum ${\nu^{\prime }}_{eL}=\sum_{i}V_{ei}^{\left(\nu \right)}\nu_{iL}$ we kept only the *ν*_1_−term for simplicity. The quantity *ω_B_* contains the product of the elements of the mixing matrices involved. From (28) we have

$$Im{\bar{a}}_{LS}=\sum_{j=2,3}\frac{Im(\lambda_{1j1}{\lambda^{\prime }}_{j11}^{*}\omega_{B})}{4m_{{\tilde{e}}_{j}L}^{2}}\left(\frac{\sqrt{2}}{G_{F}V_{ud}}\right).$$ (29)

CP-violation can arise in (28) from complex *λ*_1_*_j_*_1_ and *λ′_j_*_11_, and also from complex *ω_B_*.

In the following we shall assume for simplicity that *λ*_1__*j*1_ and ${\lambda^{\prime }}_{j11}^{*}$ are real, and that mixing for the right-handed fields and for *u_L_*-type quarks can be neglected. Then $Im(\lambda_{ij1}{\lambda^{\prime }}_{j11}^{*}\omega_{B})=\lambda_{1j1}{\lambda^{\prime }}_{j11}\;\cos\;\theta_{\nu }\;\sin\;\phi_{B}$, is a mixing angle in $V_{ei}^{\left(\nu \right)}$, and $e^{i\phi B}$ is a CPV phase.

In deriving limits on $Im{\bar{a}}_{LS}$ we shall assume (to preclude additional constraints to apply and the possibility of a cancellation in $Im{\bar{a}}_{LS}$) that only one of the products $\lambda_{1j1}{\lambda^{\prime }}_{j11}^{*}$ has a significant size at a time.

The limits on $\left|Im{\bar{a}}_{LS}\right|$ in Eq. (29) implied by limits on the individual coupling constants *λ*_1_*_j_*_1_ and *λ′_j_*_11_, derived from various processes [35], are not better than a few times 10^−2^. A stringent limit

$$\left|Im{\bar{a}}_{\mathrm{LS}}\right|<4\times 10^{-4}$$ (30)

on $Im{\bar{a}}_{LS}$ comes from the ratio *R*_π_ = Γ(π → e*ν*_e_)/Γ(π → *µν_µ_*) [37]. This limit arises because the *a_LP_* -component of (28) contributes to π → e*ν*_e_, and *a_LS_* = *a_LP_*.

Potentially the strongest limits on $Im{\bar{a}}_{LS}$ come from experimental bounds on *P,T*-violating electron-quark (*e* − *q*) interactions. As pointed out in Ref. [38], electroweak radiative corrections to scalar, pseudoscalar, and tensor interactions of any origin induce contributions to *P,T*-violating *e* − *q* interactions. For the Hamiltonian (28) this interaction is of the form^13^

$$H_{ed}=\frac{G_{F}}{\sqrt{2}}k_{Sd}\;(\bar{e}i\gamma_{5}e\bar{d}d-\bar{e}e\bar{d}i\gamma_{5}d)$$ (31)

with

$$k_{Sd}\equiv {\left(k_{Sd}\right)}_{r}=-4\rho V_{ud}Im{\bar{a}}_{LS,}$$ (32)

where $\rho =(\alpha /4\pi )\;\ln\;(\Lambda^{2}/m_{W}^{2})$; *Λ* is a cut-off parameter. Taking conservatively, as in Ref. [38], $\ln\;(\Lambda^{2}/m_{W}^{2})=1$, one has $\rho ≃6\times 10^{-4}$.

In addition to (*k_Sd_*)_r_, there is also a tree-level contribution (*k_Sd_*)*_t_* to *k_Sd_*, governed by the same products $\lambda_{1j1}{\lambda^{\prime }}_{j11}^{*}$ as $H_{\beta }^{\left(j\right)}$ in Eq. (28). This is a consequence of gauge invariance of W_Ł_ before symmetry breaking. (*k_Sd_*)*_t_* is given by

$${\left(k_{Sd}\right)}_{t}=-\frac{Im(\lambda_{1j1}{\lambda^{\prime }}_{j11}^{*}\omega_{e})}{2m_{{\tilde{\nu }}_{j}L}^{2}}\left(\frac{\sqrt{2}}{G_{F}}\right),$$ (33)

where *ω*_e_ contains the product of the appropriate mixing matrix elements. Under our simplifying assumptions $Im(\lambda_{1j1}{\lambda^{\prime }}_{j11}^{*}\omega_{e})=\lambda_{1j1}{\lambda^{\prime }}_{j11}\;\cos\theta_{e}\;\sin\phi_{e}$. It can be shown that the phases e*^iφB^* and e*^iφe^* are in general different.

The total contribution to *k_Sd_* can be written as

$$k_{Sd}={\left(k_{Sd}\right)}_{r}+{\left(k_{Sd}\right)}_{t}=-\left(4\rho +2\frac{m_{\tilde{e}jL}^{2}}{m_{\tilde{\nu }jL}^{2}}\frac{\cos\theta_{e}\;\sin\phi_{e}}{\cos\theta_{\nu }\;\sin\phi_{B}}\right)V_{ud}\;Im{\bar{a}}_{LS}.$$ (34)

It can be shown that $m_{\tilde{e}j}^{2}/m_{\tilde{\nu }jL}^{2}≲4$.

The best limit on *k_Sd_* comes from the EDM of the Tl atom. The experimental limit on d(Tl) [40] implies^14^ |*k_Sd_*| < 4.5 × 10^−8^, so that

$$\left|Im{\bar{a}}_{LS}\right|<\frac{4.5\times 10^{-8}}{V_{ud}\left(4\rho +2\frac{m_{\tilde{e}jL}^{2}}{m_{\tilde{\nu }jL}^{2}}\frac{\cos\theta_{e}\;\sin\phi_{e}}{\cos\theta_{\nu }\;\sin\phi_{B}}\right)}.$$ (35)

*(k_sd_*)_r_ alone would give a limit $\left|Im{\bar{a}}_{LS}\right|<2\times 10^{-5}$. The upper limit on $\left|Im{\bar{a}}_{LS}\right|$ could be larger than 2 × 10^−5^ if $2(m_{\tilde{e}j}^{2}/m_{\tilde{\nu }jL}^{2})(\;\cos\theta_{e}\;\sin\phi_{e}/\;\cos\theta_{\nu }\;\sin\phi_{B})$ is small, and there is a cancellation between the two terms in the denominator in Eq. (35). To allow $\left|Im{\bar{a}}_{LS}\right|≃10^{-2}$ this cancellation would have to occur through 3 orders of magnitude! The bound [Eq. (30)] from *R*_π_ would however still remain. This bound would become weaker if there is some cancellation between the contributions to π → e*ν*_e_ and π → *µν_µ_*. A contribution to π → *µν_µ_* is present in the model.

For *R_t_* in the other extensions of the SM the situation is similar to the one in the (RMSSM), provided that the associated *P,T*-violating *e − q* interaction involves only the *d*-quark. If *e − u* interactions are present, a cancellation between the radiative and tree-level contributions cannot be arranged in more than one atomic EDM. Stringent limits, albeit not as strong as from *d*(Tl), then persist [32].

## 5. Conclusions

In this talk we have discussed tree-level contributions to *D_t_* and *R_t_* in extensions of the SM. A major question is what experimental sensitivities are required to obtain new information on the new interactions involved.

For *D_t_/a* (Eq. 8) in left-right symmetric and exotic fermion models the EDMs of the neutron and of mercury set upper limits about two orders of magnitude below the present direct limits. Since the limits from the EDMs have uncertainties (from the calculation of the hadronic matrix elements and for *d*(Hg) also from nuclear structure) which are difficult to asses, the possibility that *D_t_/a* is larger cannot be ruled out. For *D_t_* mediated by leptoquark exchange the conclusion based on dimensional estimates of the electron EDM and the electric and chromoelectric quark dipole moments is that *D_t_/a* can be as large as the present experimental limit on *D_t_/a*.

For *R_t_* in neutron decay (Eq. 19) experimental limits on atomic EDMs set limits which are below the level where *R_t_* can be probed. Nevertheless, the possibility that *R_t_* is larger, even as large as ∼10^−2^, cannot be completely ruled out. This would require some very fine-tuned cancellations between the contributions to *P,T*-violating *e − q* interactions and in the ratio Γ(π → *eν*_e_) / Γ(π → *µvµ*).

## Footnotes

1 For a review, see Ref. [1].

2 For a review, see Ref. [4].

3 For a review, see Ref. [5].

4 It should be noted however that a disagreement has been found between experiment and the SM prediction in B° → *K_S_ϕ* decay [6]. See however Ref. [7].

5 For a review, see for example Ref. [8].

6 Our metric, *γ* matrices and *σ_λµ_* are the same as in Ref. [10].

7 Couplings involving neutrino states other than $\nu_{e}^{\left(L\right)}$ are possible, but for those in most cases additional constraints apply. Also, the choice $\nu_{e}^{\left(L\right)}$ in Eq. (3) guarantees for *D_t_* and *R_t_* maximal overlap in the interference with the SM amplitude.

8 For a recent review of possible new interactions in beta decay see Ref. [12].

9 Ref. [24]. See also Ref. [12].

10 Ref. [25]. Contact beta decay interactions have been discussed in Ref. [12].

11 For the Schiff moment and the EDM of mercury we used the results obtained in Refs. [28] and [29], respectively.

12 For reviews, see Refs. [33], [34], and [35].

13 Ref. [38]. See also Ref. [39].

14 Ref. [39]. See also Ref. [41].
